# Migration modulates the prevalence of ASD and ADHD: a systematic review and meta-analysis

**DOI:** 10.1186/s12888-022-04037-4

**Published:** 2022-06-13

**Authors:** Xuping Gao, Yilu Zhao, Ning Wang, Li Yang

**Affiliations:** 1 Department of Child & Adolescent Psychiatry, Peking University Sixth Hospital (Institute of Mental Health), National Clinical Research Center for Mental Disorders and NHC Key Laboratory of Mental Health (Peking University Sixth Hospital), 51, Huayuan Bei Road, Haidian District, Beijing, 100191 PR China; 2 Department of Public Health and Preventive Medicine, School of Medicine, Jinan University, No.601 Huangpu Road West, Guangzhou, 510632 Guangdong PR China

**Keywords:** Migration, Autism spectrum disorder, Attention-deficit/hyperactivity disorder, Meta-analysis

## Abstract

**Background:**

Migration has been implicated as a risk factor for autism spectrum disorder (ASD) and attention-deficit/hyperactivity disorder (ADHD), but evidence is still limited and inconsistent. We aim to investigate the relationship between migration status and risk of ASD and ADHD.

**Methods:**

Electronic databases including PubMed, EMBASE, Web of Science, and PsychINFO were searched to identify observational studies on this topic, from inception to February 2021. Random-effects meta-analysis models were used to pool the summary odds ratio (OR) and 95% confidence interval (95% CI), and subgroup analyses were conducted to detect possible discrepancies in associations. Certainty of evidence was assessed as per the Grading of Recommendations, Assessment, Development and Evaluations (GRADE) guidelines.

**Results:**

A total of 13 studies (6,532,546 participants) for ASD, five studies (2,875,070 participants) for ADHD, and six studies (31,158 participants) for hyperactivity were included. Overall, the pooled results indicated that migration was associated with increased risk of ASD (pooled OR: 1.32; 95% CI: 1.07–1.63; *P* for Z test = 0.010), but no association was found between migration and ADHD (pooled OR: 0.84; 95% CI: 0.53–1.32; *P* for Z test = 0.452) or hyperactivity (pooled standardized mean difference: -0.073; 95% CIs: − 0.383–0.236; *P* for Z test = 0.642). Subgroup analyses further demonstrated that maternal migration was ASD risk factor (pooled OR: 1.49; 95% CI: 1.19–1.87), and migrant children were more likely to develop ASD with comorbid intellectual disability (ID) (pooled OR: 1.21, *P* for interaction = 0.006) than ASD without ID. After standardized the origin of migrants, European migrant children from Americas were at higher risk of ASD and ADHD (pooled OR were 4.13 and 1.26), and increased ASD risk was also observed in African children (pooled OR: 2.72). The GRADE of evidence was very low.

**Conclusions:**

Maternal migration is a risk factor for ASD, and migrant ASD children are more likely comorbid ID. The role of migration on ADHD remains controversial, more studies are needed to assess the association between migration status and ADHD. Health care practitioners should consider screening and providing extra resources for migrant children.

**Supplementary Information:**

The online version contains supplementary material available at 10.1186/s12888-022-04037-4.

## Introduction

Over the past two decades, the number of international migrants have grown robustly from 153 million in 1990 to 281 million in 2020, and much of this increase was due to labour, family migration, or humanitarian crises in many parts of the world [1]. It is estimated that children and adolescents comprise 14.6% of all international migrants (first-generation migrants) [1], but the underlying greater number of migrant offspring (second-generation migrants) has not been well evaluated. On the one hand, supported by appropriate policies, migration may be expected to facilitate access to better health care and education for migrant children and adolescents [2]. However, on the other hand, socioeconomic barriers occur during migrant integration process in host societies which may have adverse effects on the migrant children and adolescents, especially on mental health [3, 4]. With an increasing amount of migrants, migration background has become an important variable when studying mental health from a public health perspective [5].

Autism spectrum disorder (ASD) and attention-deficit/hyperactivity disorder (ADHD) are highly correlated childhood-onset mental disorders, with prevalence of 1.85 and 5.29%, respectively [6–8]. The core symptoms of ASD (social communication and interaction impairments, and restricted repetitive behaviours, interests and activities) and ADHD (age-inappropriate inattentiveness, impulsivity, and hyperactivity) present in early childhood, and often persist into adulthood [9–11]. Poor parental socioeconomic status is associated with increased risk of ASD and ADHD [12, 13]. Migrant children may have parental socioeconomic disadvantage, have experienced severe premigration trauma, and could face ongoing chronic stress and traumatization after migration, including family separation, detention and parental deportation [14]. Migration status is a suggestive risk factor for heterogeneous psychiatric conditions [15], but the role of migration in ASD and ADHD remains uncertain [16–19].

A pervasive review of risk factors of autism listed maternal migration as suggestive evidence [20]. Pooling five studies, the meta-analysis found a marginal association between maternal migration and autism with high heterogeneity [summary effect estimate: 1.28, 95% confidence interval (CI): 0.99–1.65], and significant association was observed in Nordic studies (summary effect estimate: 1.58, 95%CI: 1.14–2.19) [21]. According to a Netherlands study, children with paternal migration were at lower risk of ASD [rate ratio (RR): 0.60, 95 95%CI: 0.50–0.90] [22]. However, this finding was not confirmed in other studies [18, 23]. The certain effect of migration status on ASD may differ across parental history of migration, generation of migration, as well as origin and destination area of migrant, which needs systematic assessments [20, 21, 24].

Measured by the Strengths and Difficulties Questionnaire (SDQ), Derluyn et al. [25] found migrant children might have lower hyperactivity score comparing to non-migrant children. But other studies did not detect any significant difference in hyperactivity score stratified by migration status [26, 27]. Result from 17,461 participates, Huss et al. suggested families with history of migration had fewer ADHD diagnoses among children and adolescents [odds ratio (OR): 0.50, 95%CI: 0.32–0.78] [28]. On the contrary, study by Lehti et al. observed a significantly increased likelihood of being diagnosed with ADHD among children who have two immigrant parents (OR: 4.7, 95% CI: 3.4–6.6) [29]. Current evidence about migration status associated with ADHD and related symptom among children and adolescents remains inconsistent.

ASD and ADHD are the most devastating mental disorders of childhood in terms of prevalence, morbidity, outcome, impact on the family, and cost to society. Investigating the relationship between migration and these disorders can help to provide better mental health service for this population. Therefore, we conducted this systematic review and meta-analysis to evaluate the current epidemiological evidence about migration status and risk of ASD and ADHD.

## Methods

### Search strategy and selection criteria

In this systematic review and meta-analysis, we searched PubMed, EMBASE, Web of Science Core Collection, and PsycINFO, for studies published in English between database inception and February, 2021. We included epidemiological studies that measured the association between migration status and risk of ASD (including autism, Asperger’s syndrome, autistic disorder, autism spectrum disorder, or pervasive developmental disorder [PDD]) or ADHD in children and adolescents (0–19 years) and provided risk estimates including OR, RR, or hazard ratio (HR) with 95% CIs. Given the limited evidence about ADHD, we further included studies comparing ADHD symptom scores between migrant and non-migrant children as a supplementary analysis. Migrant children were defined as 1) the children who moving away from the place they live, or 2) the children had one or more parent born aboard. We included internal and international children migration, defined as migration within and beyond a country’s borders, respectively. We included children migration for any reason, such as employment (labour migration), armed conflict or disaster (forced migration). The comparator group was non-migrant (native) children. Conference abstracts and review articles were excluded. For overlapping data, study with the largest dataset was reserved. The search strategy and full list of search terms used were provided in the supplementary material (Appendix 1). We identified additional studies by manual searching the reference list of included studies. All titles, abstracts, and full-text articles were screened independently by two investigators. Disagreements were resolved through discussion and adjudication by a third investigator, as needed.

The present study was conducted and reported according to the Preferred Reporting Items for Systematic Reviews and Meta-Analyses 2020 guidelines (Supplementary Table 1) [30]. This review was not registered, and the protocol was available from the corresponding author upon request.

### Data extraction and quality assessment

Two investigators independently extracted data and evaluated the quality of each study. Disagreements were resolved through discussion. The following data were extracted from each publication: first author, year of publication, location, number of participants, age at diagnosis, definition and type of migration, outcome and assessment tool, maximally adjusted ORs, RRs or HRs with 95% CIs and corresponding adjusted covariates, and mean symptom score with SD.

The methodological quality of included studies was assessed using the Newcastle-Ottawa Quality Assessment Scale (NOS) [31]. Study quality was evaluated based on the selection, comparability, exposure (for case-control studies) or outcome (for cohort studies) with a maximum score of 9. Study with a score ≥ 7 was considered as a high-quality study.

### Statistical analysis

We investigated the associations between the migration status (migrant group versus non-migrant group) and risk of ASD or ADHD as the main analysis and compared the symptom score between groups as supplementary analysis. The priori Der-Simonian and Laird random-effects model was chosen because it accounted for both within- and between-study heterogeneity [32, 33]. We estimated pooled ORs with 95% CIs for the risk of ASD and ADHD, and standardized mean differences (SMDs) with 95% CIs for symptom score comparisons. Between-study heterogeneity was tested using I^2^ statistic, an I^2^ ≥ 50% was considered to represent high heterogeneity [34].

If a study did not provide the overall risk estimate for migrant children being diagnosed as ASD or ADHD compared to non-migrant control (or symptom score differences between groups), we firstly pooled a within-study OR (SMD) for overall estimate. Subsequently, subgroup analyses were carried out stratified by ASD comorbid intellectual disability (yes or no), migration generation (1st-generation or 2nd-generation), parental country of birth (foreign-born mother, foreign-born father, or both parents foreign-born), the origin of European migrant children (classified according to the World Health Organization, including Africa, Americas, Eastern Mediterranean, Europe, South-East Asia, and Western Pacific), and NOS score (< 7 or ≥ 7).

Publication bias was detected using Egger’s and Begg’s linear regression asymmetry tests [35], and identified publication bias was adjusted using trim-and-fill method [36]. We also conducted influence analyses to assess the effect of each individual study on the summary estimates. All analyses were conducted using Stata software version 11.0 (StataCorp, College Station, TX, USA). *P* value < 0.05 inferred the statistical significance for all analyses, except for the Egger’s and Begg’s tests (*P* value < 0.10) due to their low statistical power.

The Grading of Recommendations, Assessment, Development and Evaluations (GRADE) guidance was used to rate the certainty of evidence [37] and presented in Appendix 2.

## Results

**Fig. 1:**
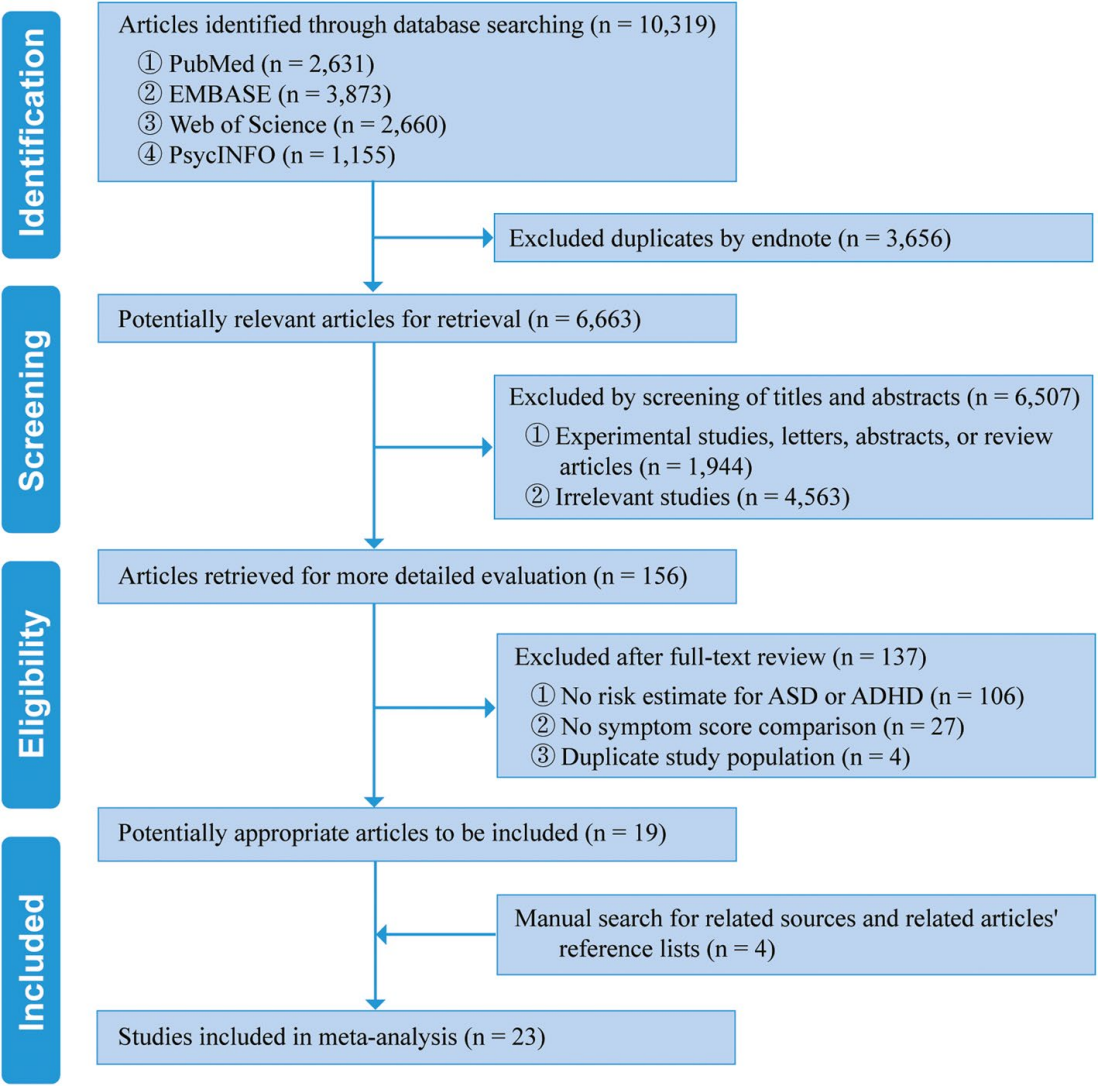
Flowchart of study selection in the current systematic review and meta-analysis. Abbreviations: ADHD, attention-deficit/hyperactivity disorder; ASD, autism spectrum disorder

A total of 10,319 records were identified by retrieving electronic databases from inception to February 2021, of which 6663 were retained after manual removal of duplicates. Through titles and abstracts sifting, 6507 irrelevant articles were removed. After reviewed full texts of remaining articles, 127 records including four studies using duplicate population [38–41] was further excluded. To guarantee the comprehensiveness of current study, four eligible articles were further included through manual search and updated search. Finally, 23 eligible studies were included in this systematic review and meta-analysis (Fig. 1).

The characteristics of included studies were described in Table 1 and Supplementary Table 2. Eligible studies were published between 2002 and 2021, including nine cohort studies [19, 22, 26, 41, 42, 44, 46, 49, 50], nine case-control studies [18, 23–25, 27, 29, 43, 45, 47], and five cross-sectional studies [28, 48, 51–53]. All studies were carried out in developed countries, and mainly concentrated in Europe. The ASD and ADHD cases were mainly diagnosed according to International Classification of Diseases, 10th Edition (ICD-10) or the Diagnostic and Statistical Manual of Mental Disorders, 4th Edition (DSM-IV). Comparative SDQ studies screening ADHD-related problems were included as supplementary analysis. The NOS score of included studies were relatively high (20/23 reached 7 score), suggesting the high quality of this study (Supplementary Tables 3 and 4). Based on GRADE guidance, the certainty of evidence was very low.

**Table 1:** Characteristics of the included studies

First author (year)	Country	Design	No. participants	Definition and type of migration	Original area or country	Outcome	Outcome assessment	Effect indicator	NOS score
**Risk of ASD**									
Croen et al. (2002) [42]	U.S.	Cohort	3,551,306	Maternal country of birth	California Other U.S. state Mexico Other	Autism	DSM-III-R and IV	RR	9
Hultman et al. (2002) [43]	Sweden	Nested case-control	Case: 408 Control: 2040	Maternal country of birth	Nordic Europe and North America Outside Europe and North America	Infantile autism	ICD-9	OR	7
Lauritsen et al. (2005) [44]	Denmark	Cohort	943,664	Maternal country of birth	Denmark Scandinavia and Europe (except Denmark) Outside Europe	Childhood autism or atypical autism	ICD-10	RR	9
Maimburg et al. (2006) [23]	Denmark	Case-control	Case: 473 Control: 4730	Parent with foreign citizenship	–	Infantile autism	ICD-8 and ICD-10	OR	7
Williams et al. (2008) [45]	Australia	Case-control	Case: 182 Control: 85,685	Maternal country of birth	–	Autistic disorder	DSM-IV	OR	6
Keen et al. (2010) [46]	U.K.	Cohort	Lambeth: 137 cases Wandsworth: 258 cases	Maternal region of birth	U.K. Other Europe Africa Caribbean Asia Elsewhere	ASD	ICD-10	RR	7
Haglund et al. (2011) [47]	Sweden	Case-control	Case:157 (autism) 93 (Asperger) Control: 68,964	Maternal country of birth	–	Autism or Asperger’s syndrome	DSM-III and IV, ICD-10, and Gillberg criteria.	OR	7
Magnusson et al. (2012) [24]	Sweden	Nested case-control study	Case: 3918 Control: 40,045	Parental country of birth	Sweden Northern Africa Eastern Africa Other African Northern America Latin America/Caribbean Southern Asia Western Asia Other Asian Northern Europe Eastern Europe Southern Europe Western Europe	ASD, ASD comorbid intellectual disability or not	Structured diagnostic assessments. Wechsler Intelligence Scale for Children, Wechsler Preschool and Primary Scale of Intelligence, Snijders-Oomen Non-Verbal Intelligence Test (Revised), and Leiter	OR	8
Lehti et al. (2013) [18]	Finland	Nested case-control study	Case: 1132 Control: 4515	Parental country of birth	Finnish Western countries Former Soviet Union and former Yugoslavia Sub-Saharan Africa North Africa, Middle East Asia	Autism	ICD-9 and ICD-10	OR	8
Singh et al. (2013) [48]	U.S.	Cross-sectional	91,532	Children with one or both immigrant parents (born outside the U.S.)	U.S.-born non-Hispanic white children Hispanic immigrant children Non-Hispanic white immigrant children Non-Hispanic black immigrant children Asian immigrant children Other immigrant children	ASD, ADHD	Question based	OR	7
van der Ven et al. (2013) [22]	Netherlands	Cohort	106,953	Paternal country of birth	Netherlands Developing countries Turkey Morocco Suriname and Dutch Antilles Other Developed countries	ASD, autistic disorder, Asperger syndrome and Pervasive Developmental Disorder Not Otherwise Specified (PDDNOS)	DSM-IV	RR	8
Becerra et al. (2014) [49]	U.S.	Cohort	1,626,354	Parental country of birth	U.S.-born Foreign-born	Autistic disorder and Autistic disorder comorbid intellectual disability	DSM- IV-R and ICD-9-CM as reported on the DDS Client Development Evaluation Report (CDER).	RR	9
Abdullahi et al. (2019) [41]	Australia	Cohort	765,064	Maternal country of birth	Australian-born mothers Foreign-born mothers from low-income countries Foreign-born mothers from lower-middle-income countries Foreign-born mothers from upper-middle-income countries Foreign-born mothers from high-income countries	ASD with/without intellectual disability	NR	RR	7
**Risk of ADHD**									
Huss et al. (2008) [28]	German	Cross-sectional	17,461	Children with migration history	–	ADHD and potential ADHD	ICD-10 and DSM-IV	OR	7
Lehti et al. (2016) [29]	Finland	Nested case-control study	Case: 10,409 Control: 39,124	Immigrant parents were defined as those who were born abroad and not native Finnish speakers.	Finland Western countries Former Soviet Union and former Yugoslavia Sub-Saharan Africa North Africa, Middle East Asia Latin America	ADHD	ICD-9 and ICD-10	OR	9
Cotter et al. (2019) [50]	Ireland	Cohort	8568	Children without Ireland citizenship	–	Hyperactivity	SDQ	OR	6
Osooli et al. (2021) [19]	Sweden	Cohort	2,707,976	First-generation migrant children were born abord, second-generation migrant children were born in Sweden with foreign-born parents	Finland Other Scandinavian countries Eastern Europe West, Central, and South Europe East Asia and the Pacific Central-South Asia Middle East and North Africa Sub-Saharan Africa North America Latin America and the Caribbean	ADHD	DSM-V and ICD-10	HR	9
**Hyperactive score (SDQ)**									
Leavey et al. (2004) [51]	U.K.	Cross-sectional	Case: 206 Control: 123	Children foreign born	U.K.-born Foreign born	Hyperactivity score	SDQ self-report version	Mean (SD)	6
Derluyn et al. (2007) [25]	Belgium	Case-control	Case: 1219 Control: 607	Migrant status	Non-migrant adolescents Migrant adolescents	Hyperactivity score	SDQ self-report version	Mean (SD)	7
Holling et al. (2008) [52]	German	Cross-sectional	Case: 2349 Control: 12,460	Children with one or both immigrant parents (born outside the Germany)	–	Hyperactivity score	SDQ parent version	Mean (SD)	7
Sagatun et al. (2008) [26]	Norway	Cohort	2455	Parental country of birth	–	Hyperactivity score	SDQ self-report version	Mean (SD)	6
Alonso-Fernandez et al. (2017) [27]	Spain	Case-control	Case: 415 Control: 830	The subjects chose the “foreigner” option as an answer to the question “What is the nationality of …?”	–	Hyperactivity score	SDQ	Mean (SD)	7
McMahon et al. (2017) [53]	Austria Estonia France Germany Hungary Ireland Italy Romania Slovenia Spain	Cross-sectional	Case:1900 Control: 9018	First-generation migrant children were born aboard, second-generation migrant children were domestically born with foreign-born parents	Non-migrants First-generation migrants (European origin) Second-generation (European origin) First-generation migrants (non-European origin) Second-generation (non-European origin)	Hyperactivity score	SDQ self-report version	Mean (SD)	8

*Abbreviations*: *ADHD* Attention-deficit/hyperactivity disorder, *ASD* Autism spectrum disorder, *CI* Confidence interval, *DSM* Diagnostic and Statistical Manual of Mental Disorders, *HR* Hazard ratio, *ICD* International Classification of Diseases, *NOS* Newcastle–Ottawa scale, *NR* Not report, *OR* Odds ratio, *RR* Risk ratio, *SD* Standard deviation, *SDQ* Strengths and Difficulties Questionnaire

### Autism spectrum disorder among migrant children

**Fig. 2:**
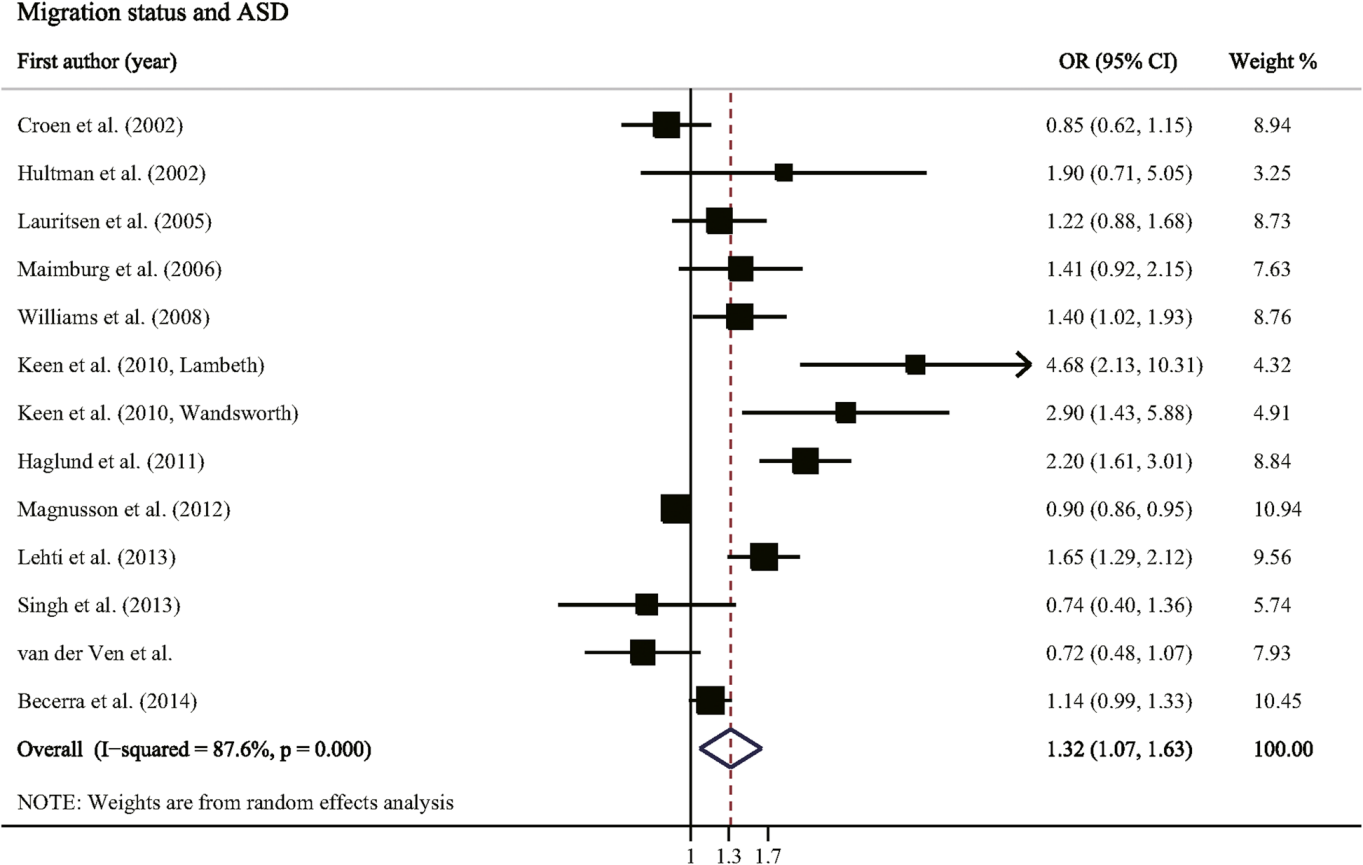
Forest plot of the association between migration status and risk of ASD. Error bars indicate 95% confidence intervals**.** Abbreviations: ASD, autism spectrum disorder; CI, confidence interval; OR, odds ratio

13 studies involving 6,532,546 participants reported the association between migration status and risk of ASD. Compared to non-migrant children, the pooled OR for ASD among migrant children was 1.32 (95% CI: 1.07–1.63, *P* = 0.010, Table 2 and Fig. 2) with a high heterogeneity (I^2^ = 87.6%, *P* < 0.001).

**Table 2:** Associations between migration status and risk of ASD

Groups	No. studies	No. participants	Pooled ORs (95% CIs)	***P*** for Z test	Heterogeneity		***P*** for interaction
					I^**2**^ (%)	***P***	
**Overall migration**	13	6,532,546	**1.32 (1.07, 1.63)**	0.010	87.6	< 0.001	
**Diagnostic subgroup**							
Autism	7	6,220,489	**1.26 (1.06, 1.50)**	0.010	57.0	0.030	0.583
Autism spectrum disorder	6	312,057	1.46 (0.89, 2.40)	0.133	91.4	< 0.001	
**Comorbid intellectual disability**							**0.006**
Yes	4	2,435,381	**1.21 (1.09, 1.35)**	0.001	4.6	0.370	
No	3	809,027	0.68 (0.46, 1.01)	0.057	84.0	0.002	
**Parental country of birth**							0.514
Foreign-born mother	11	6,334,601	**1.49 (1.19, 1.87)**	0.001	81.9	< 0.001	
Foreign-born father	2	10,850	1.19 (0.86, 1.65)	0.302	0.0	0.617	
Both parents foreign-born	2	49,610	1.24 (0.63, 2.43)	0.541	90.9	0.001	
**Migration type** ^**a**^							**0.016**
Africa to Europe	4	50,005	**2.72 (1.25, 5.89)**	0.011	93.6	< 0.001	
Americas to Europe	4	50,005	**4.13 (1.26, 13.50)**	0.019	96.6	< 0.001	
Eastern Mediterranean to Europe	2	49,610	0.88 (0.61, 1.26)	0.489	0.0	0.867	
Europe to Europe	5	993,669	1.20 (0.97, 1.50)	0.097	41.7	0.143	
South-East Asia to Europe	3	44,358	1.80 (0.71, 4.56)	0.213	92.7	< 0.001	
Western Pacific to Europe	1	43,963	0.90 (0.60, 1.30)	–	–	–	
**NOS score**							0.739
< 7	1		1.40 (1.00, 1.90)	–	–	–	
≥ 7	12		**1.31 (1.05, 1.64)**	0.017	88.0	< 0.001	
**Influence analyses** ^**b**^							
Minimal	–	6,463,332	**1.24 (1.01, 1.51)**	0.036	84.4	< 0.001	
Maximal	–	6,425,593	**1.39 (1.11, 1.73)**	0.004	88.3	< 0.001	

^a^The number of participants was calculated based on the total population of included studies in each group

^b^Influence analysis was conducted by eliminating one study at a time; excluded study by Haglund et al. [47] for minimal pooled ORs, and excluded study by van der Ven et al. [22] for maximal pooled ORs

*Abbreviations*: *CI* Confidence interval, *NOS* Newcastle-Ottawa scale, *OR* Odds ratio

Subgroup analyses indicated that migrant children were more likely to develop ASD with comorbid intellectual disability (pooled OR: 1.21, *P* for interaction = 0.006). Stratified by parental country of birth, migrant children with a foreign-born mother had a significant increased risk of ASD (pooled OR: 1.49; 95% CI: 1.19–1.87), but no significant result was observed among migrant children with foreign-born father or both parents foreign-born (*P* for Z test > 0.05). Migrant children moving from Africa and Americas were at significantly increased risk of ASD (pooled OR were 2.72 and 4.13, *P* for interaction = 0.016). No significant interaction was observed when stratified by diagnostic subgroup or NOS score (*P* for interaction were 0.583 and 0.739).

### Attention-deficit hyperactivity disorder among migrant children

**Fig. 3:**
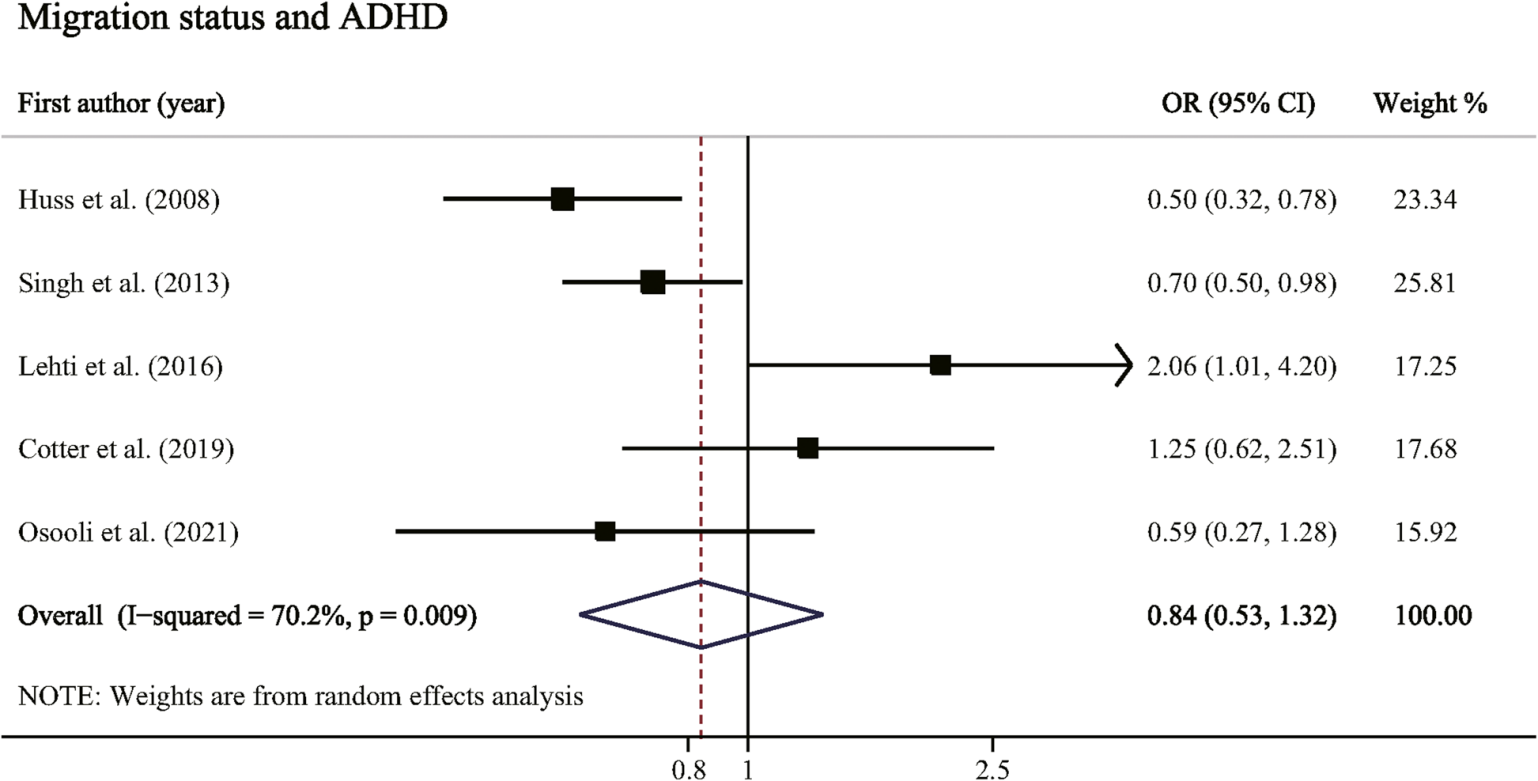
Forest plot of the association between migration status and risk of ADHD. Error bars indicate 95% confidence intervals**.** Abbreviations: ADHD, attention-deficit/hyperactivity disorder; CI, confidence interval; OR, odds ratio

Five studies involving 2,875,070 participants reported the association between migration status and risk of ADHD. The pooled OR for overall migration was 0.84 (95% CI: 0.53–1.32, *P* = 0.452, Table 3 and Fig. 3) with a high heterogeneity (I^2^ = 70.2%, *P* = 0.009). Comparing SDQ hyperactivity score between migrant children and non-migrant control, supplementary meta-analysis indicated there was no significant difference between groups (*P* for Z test = 0.642, Supplementary Table 5 and Supplementary Fig. 1).

**Table 3:** Associations between migration status and risk of ADHD

Groups	No. studies	No. participants	Pooled ORs (95% CIs)	***P*** for Z test	Heterogeneity		***P*** for interaction
					I^**2**^ (%)	***P***	
**Overall migration**	5	2,875,070	0.84 (0.53, 1.32)	0.452	70.2	0.009	
**Migration generation**							0.726
1st generation	2	2,716,544	0.67 (0.22, 2.06)	0.488	89.2	0.002	
2nd generation	4	2,866,502	0.83 (0.54, 1.28)	0.404	74.3	0.009	
**Parental country of birth**							**0.008**
Foreign-born mother	2	2,757,509	**0.92 (0.88, 0.95)**	< 0.001	0.0	0.467	
Foreign-born father	2	2,757,509	**1.58 (1.12, 2.23)**	0.009	94.5	< 0.001	
Both parents foreign-born	2	2,757,509	1.50 (0.16, 14.10)	0.724	98.9	< 0.001	
**Migration type** ^**a**^							**< 0.001**
Africa to Europe	2	2,757,509	1.81 (0.30, 10.94)	0.516	97.3	< 0.001	
Americas to Europe	2	2,757,509	**1.26 (1.14, 1.40)**	< 0.001	0.0	0.642	
Eastern Mediterranean to Europe	2	2,757,509	1.29 (0.37, 4.50)	0.689	95.7	< 0.001	
Europe to Europe	2	2,757,509	1.16 (0.61, 2.20)	0.650	96.9	< 0.001	
South-East Asia to Europe	2	2,757,509	0.89 (0.29, 2.77)	0.838	91.8	< 0.001	
Western Pacific to Europe	1	2,707,967	**0.40 (0.27, 0.61)**	< 0.001	96.6	< 0.001	
**NOS score**							0.271
< 7	1	8568	1.25 (0.63, 2.51)	–	–	–	
≥ 7	4	2,866,502	0.77 (0.46, 1.29)	0.320	73.0	0.011	
**Influence analyses** ^**b**^							
Minimal	–	2,825,537	**0.68 (0.49, 0.95)**	0.024	39.3	0.176	
Maximal	–	2,857,609	0.98 (0.58, 1.68)	0.955	67.3	0.027	

^a^The number of participants was calculated based on the total population of included studies in each group

^b^Influence analysis was conducted by eliminating one study at a time; excluded study by Cotter et al. [50] for minimal pooled ORs, and excluded study by Huss et al. [28] for maximal pooled ORs

*Abbreviations*: *CI* Confidence interval, *NOS* Newcastle-Ottawa scale, *OR* Odds ratio

Subgroup analyses indicated that migrant children with a foreign-born mother were at lower risk of ADHD, on the contrary, migrant children with a foreign-born father were at higher risk of ADHD (pooled ORs were 0.92 and 1.58, *P* for interaction = 0.008). Stratified by migration type, European migrant children moving from Americas were at significantly increased risk of ADHD (pooled OR: 1.26, *P* for Z test < 0.001). No significant interaction was observed stratified by generation of migration and NOS score (*P* for interaction were 0.726 and 0.271).

### Publication bias and influence analyses

Egger’s test revealed a significant publication bias in pooled OR of ASD (Begg’s test *P* = 0.760; Egger’s test *P* = 0.011), and the funnel plot also indicated evidence of publication bias (Supplementary Fig. 2). After imputing five missing studies by using the trim-and-fill method, the recalculated pooled OR did not reach statistical significance (imputed OR: 0.98; 95% CI: 0.79–1.22; *P* for Z test = 0.855; Supplementary Fig. 3). Moreover, publication bias was also detected in foreign-born mother subgroup for ASD risk (Begg’s test *P* = 0.087; Egger’s test *P* = 0.020). After recalculated OR using the trim-and-fill method, pooled OR attenuated (imputed OR: 1.11; 95% CI: 1.03–1.21; *P* for Z test = 0.007). Influence analyses suggested the pooled ORs of ASD were stable after excluded each individual study (Table 2).

No significant publication bias was detected in pooled OR of ADHD (Begg’s test *P* = 0.806; Egger’s test = 0.360; Funnel plot was presented as Supplementary Fig. 4). Excluded study by Cotter et al., [50] influence analyses detected an significant protective effect of migration on ADHD risk (pooled OR: 0.68; 95% CI: 0.49–0.95; *P* for Z test = 0.024; Table 3).

## Discussion

This review provided integrated evidence about the association between migration status and risk of ASD and ADHD among children, and highlighted the potential psychosocial impact of migration on children’s mental health. The pooled results indicated that migrant children with foreign-born mother had an increased risk of ASD, and migrant children were more likely develop ASD with comorbid intellectual disability. Overall migration status was not associated with risk of ADHD or hyperactivity score. Based on limited evidence, subgroup analyses suggested maternal or paternal migration might have different effects on ADHD, which need further investigation. Stratified by the origin of migrants, migrant children from Americas were at higher risk of ASD and ADHD, and increased ASD risk was also observed in African migrant children. Our findings might be extrapolated to clinical trials in ASD and ADHD, allowing us to extract more interesting results. Additionally, the findings might be used to understand more about these disorders and to develop more strategies to help these children better integrate into society and help themselves as individuals.

Including 49 studies, meta-analysis by Selten et al. [54] found that migrants and their children were at increased risk of developing non-affective and affective psychotic disorder. Radua et al. [15] conducted an umbrella review to classify the factor associated with psychotic disorders, and indicated first generation immigrants, second generation immigrants, and North-African immigrants in Europe were suggestive risk factors. As a general social factor, migration background is associated with risk of ASD and ADHD, but with a small effect. Combining new evidence, our finding is consistent with pervious meta-analysis which suggested maternal migration was a risk factor for ASD [21]. Moreover, to the best of our knowledge, this study is the first one to pool evidence about migration and ADHD, and suggest migration as a potential influencing factor for ADHD.

As previous studies argued, the adverse effect of migration might associate with relative social disadvantage [55]. Due to insufficient awareness of health care policies and care systems, and limited availability of information across minority languages and financial resources, migrant children may face additional challenges in accessing health care and related services [17, 55–57]. Additionally, migrant populations have previously voiced concerns about the social stigma within their communities [58]. Social adversity in native countries may lead to major premigration stress, including psychological (perceived stress) and biological stress (biological stress responses) [59]. In addition to the severe premigration trauma, migrant children may experience ongoing chronic stress and traumatization after migration, including family separation, detention and parental deportation [14]. Potential trauma associated with the migratory trip and postmigration experience can produce a lasting epigenetic memory that may affect the behaviour and mental health status [59–61]. In addition, the dual stress of acculturation and parenting among migrant parents may also be associated with these developmental disorders in offspring [62, 63].

Nowadays, we know that ASD is a complex disorder with the interaction of both genetic and environmental factors [12, 64]. Maternal migration is associated with an increased risk of offspring ASD. This may include exposures related to migration status such as lower socioeconomic status, higher parenting stress, limited social support [12, 62]. Study by Gillberg et al. [65] suggested that migration women might not be immunized against the common infectious agents in the country where she gives birth, and therefore might be more susceptible to relatively innocuous infections which might increase the risk for ASD. Potential maternal exposure to social stressors associated with migration can also influence the development of ASD through epigenetic mechanisms, such as DNA methylation affecting gene expression [59, 66]. Moreover, our results indicated that migrant children were more likely to develop ASD comorbid intellectual disability, consistent with an early study which reported more severe intellectual impairment in migrant ASD children than natives [3]. Known ASD risk factors including intrauterine exposure to neurotoxin, alcohol, cocaine and pollutants were also associated with lower IQ scores [67]. Further confirmative studies on the role of these exposures in the co-occurring ASD and intellectual disability are needed.

For ADHD, substantial heterogeneity between studies was observed, and overall migration was not associated with ADHD or ADHD symptoms. Stratified by parental country of birth, we found that maternal and paternal migration might differ in specific effect on developing ADHD. In patriarchal societies (i.e., societies governed/run by men), social position is most of the time occupied by men compared to women, and socioeconomic precarity with its relevant social representation is usually transmitted through the paternal line [68]. Moreover, animal studies have linked sex-biased neurodevelopmental disorders with prenatal stress and traumatic experiences in early life [60, 69, 70]. Evidence supports the existence of a sensitive period of early gestation when epigenetic programming of the male germline may occur, permitting specific phenotypes to be transmitted to subsequent generations [60]. Early stress alters the profile of DNA methylation in the promoter of several candidate genes in the germline of male mice, and these alternations are also present in the brain of the offspring and correlated with changes in gene expression [69]. Traumatic stress in early life alters microRNAs (miRNAs) expression in the sperm of male mice [70, 71]. Injection of sperm RNAs from these males into fertilized wild-type oocytes can reproduce the trauma-related alterations in the resulting progeny [70, 71]. Overall, gene-environment interactions may contribute to disease inheritance across generations.

Subgroup analyses indicated that migrant children from different origin may face different disease risks. Consistent with the study by Shenouda et al. [72] which identified significant variations in ASD prevalence by race/ethnicity, and socioeconomic status. Race/ethnicity can be described as differences in nationality, culture, religion, biological factor or language [58]. Premigration sociodemographic factors may influence the associations between migration and these diseases, for instance, migrant children with lower premigration socioeconomic status are at increased risk of ASD. Our finding further confirmed the potential interaction between sociodemographic factors and the development of such mental disorders, and highlighted the importance of related research in future.

Migration status was assessed across multiple studies which achieved relatively strong statistical power detecting underlying associations; however, significant publication bias and high heterogeneity between studies were observed. Given the limitations of included studies themselves, we were unable to comprehensively assess potential factors that might influence these associations (e.g., migration generation). Therefore, the irrelevant relationships we found should be interpreted with caution and further validation is required. Other limitations of present study should also be taken into consideration. First, all eligible studies were conducted in high-income countries. Although most migrants originate from low-income or middle-income countries and relocate in search of employment opportunities [2], there are some individuals migrate to these countries as well. It’s necessary for future study evaluating the impact of migration between developing countries, as well as migration from developed to developing countries. Second, migration occurs globally but varies between regions which contributes to the heterogeneity between studies. Our results found a significant interaction between the origin of migrants and associations between migration status and risk of ASD and ADHD, underlining the importance of a detailed breakdown of origin. Third, the results differ across studies due to differences in definition of migrant children, different modelling approaches, and adjustment for different confounding factors. Hence, we performed risk estimates based on overall migration to obtain more objective assessment of the impact of migration status on ASD and ADHD. Fourth, recent studies have highlighted the importance of maternal or paternal background on diagnosis of such disorder [19, 23, 29]. Our study emphasizes the trend of related research and the need to investigate underlying mechanisms in human sample. Finally, subtle differences in diagnostic criteria of ASD or ADHD between studies was not well evaluated, which might have contributed to the variation in pooled estimates. With the increasement of studies in the future, there may be more discoveries through stratifying by refined diagnosis.

## Conclusions

Migration status had potential effect on two genetic correlated childhood-onset mental disorders. Comparing to non-migration status, maternal migration was associated with increased risk of offspring ASD. Migrant children were more susceptible to ASD with comorbid intellectual disability. Although overall migration was not associated with the risk of ADHD or hyperactive score, we observed a significant different effect of maternal and paternal migration on ADHD. Given to the children in such position might suffer social injustice and unbalanced medical resources, health care practitioners should consider screening and providing extra resources for migrant children, especially those from Americas. 

## Supplementary Information

**Additional file 1.**

## Data Availability

The datasets generated and analysed during the current study are available in published articles, DOI numbers: 10.1023/a:1015405914950; 10.1097/00001648-200207000-00009; 10.1111/j.1469-7610.2004.00391.x; 10.1111/j.1600-0447.2006.00805.x; 10.1111/j.1365-2214.2007.00796.x; 10.1192/bjp.bp.109.065490; 10.1177/1362361309353614; 10.1192/bjp.bp.111.095125; 10.1186/1471-2431-13-171; 10.1177/003335491312800606; 10.1111/acps.12054; 10.1542/peds.2013-3928; 10.1016/j.jpeds.2018.08.047; 10.1007/s00787-008-1006-z; 10.1111/jcpp.12570; 10.1017/ipm.2018.53; 10.1016/j.jpsychores.2020.110330; 10.1007/s00127-004-0724-x; 10.1007/s00787-007-0636-x; 10.1007/s00787-008-1004-1; 10.1007/s00127-007-0275-z; 10.1016/j.pedn.2017.05.005; 10.1192/bjpo.bp.117.005322.

## Abbreviations

ADHD: Attention-deficit/hyperactivity disorder
ASD: Autism spectrum disorder
CI: Confidence interval
DSM: Diagnostic and Statistical Manual of Mental Disorders
HR: Hazard ratio
ICD: International Classification of Diseases
NOS: Newcastle-Ottawa scale
NR: Not reported
OR: Odds ratio
RR: Risk ratio
SD: Standard deviation
SDQ: Strengths and Difficulties Questionnaire
