# Supplementary material for: Migration modulates the prevalence of ASD and ADHD: a systematic review and meta-analysis
Source: BMC Psychiatry. 2022 Jun 13;22:395. doi: 10.1186/s12888-022-04037-4 (PMC9195277; doi:10.1186/s12888-022-04037-4)
Supplement: Supplementary file 1 — Additional file 1. [file 12888_2022_4037_MOESM1_ESM.docx]

# Migration modulates the prevalence of ASD and ADHD: A systematic review and meta-analysis

Xuping Gao ^1, 2, #^ PhD, Yilu Zhao ^1, #^ PhD, Ning Wang ^1^ PhD, Li Yang ^1, *^ PhD

^1^ Department of Child & Adolescent Psychiatry, Peking University Sixth Hospital (Institute of Mental Health), National Clinical Research Center for Mental Disorders and NHC Key Laboratory of Mental Health (Peking University Sixth Hospital), 51 HuayuanBei Road, Beijing, 100191, PR China.

^2^ Department of Public Health and Preventive Medicine, School of Medicine, Jinan University, No.601 Huangpu Road West, Guangzhou 510632, Guangdong, PR China.

^#^ Xuping Gao and Yilu Zhao contributed equally to this work and should be considered as co-first authors.

**^*^ Reprint request and correspondence:** **Li Yang, MD, PhD,** [yangli_pkuimh@bjmu.edu.cn](mailto:yangli_pkuimh@bjmu.edu.cn).

Peking University Sixth Hospital, Peking University Institute of Mental Health, NHC Key Laboratory of Mental Health (Peking University), National Clinical Research Center for Mental Disorders (Peking University Sixth Hospital), 51, Huayuan Bei Road, Haidian District, Beijing, 100191 China

Tel.: +86 6235-0880.

**SUPPLEMENTARY MATERIAL**

# Index

**Appendix 1**. Additional details on search syntax

**Appendix 2**. GRADE of evidence

**Supplementary Table 1**. PRISMA 2020 checklist

**Supplementary Table 2**. Additional characteristics of the included studies

**Supplementary Table 3**. Quality of cohort studies included in meta-analysis according to the Newcastle-Ottawa Scale

**Supplementary Table 4**. Quality of case-control/cross-sectional studies included in meta-analysis according to the Newcastle-Ottawa Scale

**Supplementary Table 5**. Associations between migration status and hyperactive score (SDQ)

**Supplementary Fig. 1** Forest plot for standardized mean differences of hyperactivity score between migrant and non-migrant children

**Supplementary Fig. 2** Funnel plot for studies evaluating risk of ASD among migrant children

**Supplementary Fig. 3** Funnel plots for studies evaluating risk of ASD among migrant children using the trim-and-fill method

**Supplementary Fig. 4** Funnel plot for studies evaluating risk of ADHD among migrant children.

# Appendix 1. Additional details on search syntax

Search in electronic sources

- The following electronic databases were searched PubMed, EMBASE, Web of Science Core Collection, and PsycINFO.

Search syntax for each database

1. PubMed 2,631

#1 (ADHD OR attention deficit disorder with hyperactivity OR hyperkinetic syndrome OR hyperactivity disorder OR hyperactive child syndrome OR childhood hyperkinetic syndrome OR attention deficit hyperactivity disorders OR attention-deficit hyperactivity disorder OR attention deficit hyperactivity disorder OR addh OR overactive child syndrome OR attention deficit hyperkinetic disorder OR hyperkinetic disorder OR attention deficit disorder OR hyperactivity OR child attention deficit disorder OR hyperkinetic syndromes OR syndromes hyperkinetic OR hyperkinetic syndrome childhood) OR “Attention Deficit Disorder with Hyperactivity”[Mesh]

#2 (autis* OR autism* OR autistic* OR ASD OR autism spectrum disorder* OR PDD-NOS OR PDDNOS OR unspecified PDD OR pervasive developmental disorder* OR pervasive developmental disorder not otherwise specified OR asperger* OR asperger* syndrome) OR (“Autistic Disorder”[Mesh] OR “Autism Spectrum Disorder”[Mesh])

#3 ("Human Migration"[Mesh] OR "Transients and Migrants"[Mesh] OR "Emigrants and Immigrants"[Mesh]) OR (migrat* or migrant* or emigrat* or immigrat* or transient*)

#4 #1 OR #2

#5 #4 AND #3

The last search was run on February 4, 2021.

1. Embase 3,873

#1 ‘ADHD’ OR ‘attention deficit disorder with hyperactivity’ OR ‘hyperkinetic syndrome’ OR ‘hyperactivity disorder’ OR ‘hyperactive child syndrome’ OR ‘childhood hyperkinetic syndrome’ OR ‘attention deficit hyperactivity disorders’ OR ‘attention-deficit hyperactivity disorder’ OR ‘attention deficit hyperactivity disorder’ OR ‘addh’ OR ‘overactive child syndrome’ OR ‘attention deficit hyperkinetic disorder’ OR ‘hyperkinetic disorder’ OR ‘attention deficit disorder’ OR ‘hyperactivity’ OR ‘child attention deficit disorder’ OR ‘hyperkinetic syndromes’ OR ‘syndromes hyperkinetic’ OR ‘hyperkinetic syndrome childhood’ OR ‘attention deficit disorder’/exp

#2 ‘autis*’ OR ‘autism*’ OR ‘autistic*’ OR ‘ASD’ OR ‘autism spectrum disorder*’ OR ‘PDD-NOS’ OR ‘PDDNOS’ OR ‘unspecified PDD’ OR ‘pervasive developmental disorder*’ OR ‘pervasive developmental disorder not otherwise specified’ OR ‘asperger*’ OR ‘asperger* syndrome’ OR ‘Autistic Disorder’ OR ‘autism’/exp

#3 ‘migrat*’ OR ‘migrant*’ OR ‘emigrat*’ OR ‘immigrat*’ OR ‘transient*’ OR ‘migration’/exp

#4 #1 OR #2

#5 #4 AND #3

The last search was run on February 4, 2021.

1. Web of Science 2,660

#1 TOPIC: (ADHD OR attention deficit disorder with hyperactivity OR syndrome hyperkinetic OR hyperkinetic syndrome OR hyperactivity disorder OR hyperactive child syndrome OR childhood hyperkinetic syndrome OR attention-deficit hyperactivity disorder* OR attention deficit hyperactivity disorder* OR overactive child syndrome OR attention deficit hyperkinetic disorder OR hyperkinetic disorder* OR attention deficit disorder OR hyperactivity OR child attention deficit disorder OR hyperkinetic syndromes OR hyperkinetic syndrome childhood)

#2 TOPIC: (autis* OR autism* OR autistic* OR autism spectrum disorder* OR ASD OR PDD-NOS OR PDDNOS OR unspecified PDD OR pervasive developmental disorder* OR asperger* OR asperger* syndrome)

#3 TOPIC: (migrat* or migrant* or emigrat* or immigrat* or transient*)

#4 #1 OR #2

#5 #3 AND #4

The last search was run on February 4, 2021.

D. PsycINFO 1,155

#1 TX (ADHD OR attention deficit disorder with hyperactivity OR syndrome hyperkinetic OR hyperkinetic syndrome OR hyperactivity disorder OR hyperactive child syndrome OR childhood hyperkinetic syndrome OR attention-deficit hyperactivity disorder* OR attention deficit hyperactivity disorder* OR overactive child syndrome OR attention deficit hyperkinetic disorder OR hyperkinetic disorder* OR attention deficit disorder OR hyperactivity OR child attention deficit disorder OR hyperkinetic syndromes OR hyperkinetic syndrome childhood)

#2 TX (autis* OR autism* OR autistic* OR autism spectrum disorder* OR ASD OR PDD-NOS OR PDDNOS OR unspecified PDD OR pervasive developmental disorder* OR asperger* OR asperger* syndrome)

#3 TX (migrat* or migrant* or emigrat* or immigrat* or transient*)

#4 #1 OR #2

#5 #4 AND #3

The last search was run on February 4, 2021.

**Appendix 2. GRADE of evidence**

| Certainty assessment | | | | | | |  | Effect | |  | Certainty |
| --- | --- | --- | --- | --- | --- | --- | --- | --- | --- | --- | --- |
| Study design ^a^ | No. of participants (No. of studies) | Risk of bias ^b^ | Imprecision | Inconsistency ^e^ | Indirectness ^f^ | Publication bias |  | Odds ratio (95% CI) | Standardized mean differences (95% CI) |  |  |
| Migration status and risk of ASD | | | | | | | | | | | |
| Observational studies | 6,532,546 (13) | Not serious | Not serious ^c^ | Serious | Not serious | Serious ^g^ |  | 1.32 (1.07, 1.63) | - |  | ⨁◯◯◯  Very low |
| Migration status and risk of ADHD | | | | | | | | | | | |
| Observational studies | 2,875,070 (5) | Not serious | Serious ^d^ | Serious | Not serious | Not serious ^h^ |  | 0.84 (0.53, 1.32) | - |  | ⨁◯◯◯  Very low |
| Hyperactive score between migrant and non-migrant children | | | | | | | | | | | |
| Observational studies | 31,158 (6) | Not serious | Serious ^d^ | Serious | Not serious | Not serious  ^h^ |  | - | -0.073 (-0.383, 0.236) |  | ⨁◯◯◯  Very low |

**Note**:

^a^ Observational studies were rated as low certainty and could be downgraded for serious risk factors.

^b^ Quality assessments were favourable (20/23 studies reached a NOS score of 7) indicating a low risk of bias among individual studies.

^c^ Large sample size and 95% CI excluded the null value.

^d^ The 95% CI included the null value.

^e^ All I^2^ values lager than 50% indicating serious inconsistency.

^f^ No significant indirectness was observed.

^g^ Egger's test P value was 0.011.

^h^ Egger's test P values larger than 0.10.

**Abbreviations**: ADHD, attention-deficit/hyperactivity disorder; ASD, autism spectrum disorder; CI, confidence interval; NOS, Newcastle-Ottawa scale.

# Supplementary Table 1. PRISMA 2020 Checklist

| **Section and Topic** | **Item #** | **Checklist item** | **Location where item is reported** |
| --- | --- | --- | --- |
| **TITLE** | | | |
| Title | 1 | Identify the report as a systematic review. | Page 1 |
| **ABSTRACT** | | | |
| Abstract | 2 | See the PRISMA 2020 for Abstracts checklist. | Page 2 |
| **INTRODUCTION** | | | |
| Rationale | 3 | Describe the rationale for the review in the context of existing knowledge. | Page 3-4 |
| Objectives | 4 | Provide an explicit statement of the objective(s) or question(s) the review addresses. | Page 4-5 |
| **METHODS** | | | |
| Eligibility criteria | 5 | Specify the inclusion and exclusion criteria for the review and how studies were grouped for the syntheses. | Page 5 |
| Information sources | 6 | Specify all databases, registers, websites, organisations, reference lists and other sources searched or consulted to identify studies. Specify the date when each source was last searched or consulted. | Page 5 |
| Search strategy | 7 | Present the full search strategies for all databases, registers and websites, including any filters and limits used. | Appendix 1 |
| Selection process | 8 | Specify the methods used to decide whether a study met the inclusion criteria of the review, including how many reviewers screened each record and each report retrieved, whether they worked independently, and if applicable, details of automation tools used in the process. | Page 5 |
| Data collection process | 9 | Specify the methods used to collect data from reports, including how many reviewers collected data from each report, whether they worked independently, any processes for obtaining or confirming data from study investigators, and if applicable, details of automation tools used in the process. | Page 6 |
| Data items | 10a | List and define all outcomes for which data were sought. Specify whether all results that were compatible with each outcome domain in each study were sought (e.g. for all measures, time points, analyses), and if not, the methods used to decide which results to collect. | Page 6 |
|  | 10b | List and define all other variables for which data were sought (e.g. participant and intervention characteristics, funding sources). Describe any assumptions made about any missing or unclear information. | Page 6 |
| Study risk of bias assessment | 11 | Specify the methods used to assess risk of bias in the included studies, including details of the tool(s) used, how many reviewers assessed each study and whether they worked independently, and if applicable, details of automation tools used in the process. | Page 6 |
| Effect measures | 12 | Specify for each outcome the effect measure(s) (e.g. risk ratio, mean difference) used in the synthesis or presentation of results. | Page 6 |
| Synthesis methods | 13a | Describe the processes used to decide which studies were eligible for each synthesis (e.g. tabulating the study intervention characteristics and comparing against the planned groups for each synthesis (item #5)). | Page 6 |
|  | 13b | Describe any methods required to prepare the data for presentation or synthesis, such as handling of missing summary statistics, or data conversions. | Page 6 |
|  | 13c | Describe any methods used to tabulate or visually display results of individual studies and syntheses. | Page 6 |
|  | 13d | Describe any methods used to synthesize results and provide a rationale for the choice(s). If meta-analysis was performed, describe the model(s), method(s) to identify the presence and extent of statistical heterogeneity, and software package(s) used. | Page 6 |
|  | 13e | Describe any methods used to explore possible causes of heterogeneity among study results (e.g. subgroup analysis, meta-regression). | Page 7 |
|  | 13f | Describe any sensitivity analyses conducted to assess robustness of the synthesized results. | Page 7 |
| Reporting bias assessment | 14 | Describe any methods used to assess risk of bias due to missing results in a synthesis (arising from reporting biases). | Page 7 |
| Certainty assessment | 15 | Describe any methods used to assess certainty (or confidence) in the body of evidence for an outcome. | Page 7 |
| **RESULTS** | | | |
| Study selection | 16a | Describe the results of the search and selection process, from the number of records identified in the search to the number of studies included in the review, ideally using a flow diagram. | Page 8, Fig. 1 |
|  | 16b | Cite studies that might appear to meet the inclusion criteria, but which were excluded, and explain why they were excluded. | Page 8 |
| Study characteristics | 17 | Cite each included study and present its characteristics. | Page 8, Table 1, and Supplementary Table 2 |
| Risk of bias in studies | 18 | Present assessments of risk of bias for each included study. | Page 8, Supplementary Table 3 and 4 |
| Results of individual studies | 19 | For all outcomes, present, for each study: (a) summary statistics for each group (where appropriate) and (b) an effect estimate and its precision (e.g. confidence/credible interval), ideally using structured tables or plots. | Fig. 2, 3, and Supplementary Fig. 1 |
| Results of syntheses | 20a | For each synthesis, briefly summarise the characteristics and risk of bias among contributing studies. | Page 8-10 |
|  | 20b | Present results of all statistical syntheses conducted. If meta-analysis was done, present for each the summary estimate and its precision (e.g. confidence/credible interval) and measures of statistical heterogeneity. If comparing groups, describe the direction of the effect. | Table 2 and 3 |
|  | 20c | Present results of all investigations of possible causes of heterogeneity among study results. | Page 8-10, 13-14 |
|  | 20d | Present results of all sensitivity analyses conducted to assess the robustness of the synthesized results. | Page 8-9, Table 2 and 3 |
| Reporting biases | 21 | Present assessments of risk of bias due to missing results (arising from reporting biases) for each synthesis assessed. | Page 10 |
| Certainty of evidence | 22 | Present assessments of certainty (or confidence) in the body of evidence for each outcome assessed. | Appendix 2 |
| **DISCUSSION** | | | |
| Discussion | 23a | Provide a general interpretation of the results in the context of other evidence. | Page 10-12 |
|  | 23b | Discuss any limitations of the evidence included in the review. | Page 13-14 |
|  | 23c | Discuss any limitations of the review processes used. | Page 13-14 |
|  | 23d | Discuss implications of the results for practice, policy, and future research. | Page 14 |
| **OTHER INFORMATION** | | | |
| Registration and protocol | 24a | Provide registration information for the review, including register name and registration number, or state that the review was not registered. | Page 6 |
|  | 24b | Indicate where the review protocol can be accessed, or state that a protocol was not prepared. | Page 6 |
|  | 24c | Describe and explain any amendments to information provided at registration or in the protocol. | None |
| Support | 25 | Describe sources of financial or non-financial support for the review, and the role of the funders or sponsors in the review. | Page 15 |
| Competing interests | 26 | Declare any competing interests of review authors. | Page 15 |
| Availability of data, code and other materials | 27 | Report which of the following are publicly available and where they can be found: template data collection forms; data extracted from included studies; data used for all analyses; analytic code; any other materials used in the review. | Page 15 |

For more information, visit: http://www.prisma-statement.org/

# Supplementary Table 2. Additional characteristics of the included studies

| **First author (year)** | **Country** | **Design** | **Population description** | **Age at diagnosis** | **Definition and type of migration** | **Outcome** | **Adjusted Confounders** |
| --- | --- | --- | --- | --- | --- | --- | --- |
| **Risk of ASD** |  |  |  |  |  |  |  |
| Croen et al. (2002) ^1^ | US | Cohort | The study included all children born in California in 1989–1994 whose mother was a California resident at the time of delivery. | NR | Maternal birth place | Autism (excluded Asperger’s disorder, childhood disintegrative disorder, Rett’s disorder, and not otherwise specified PDD-NOS) | Sex, birth weight, plurality, birth order, maternal age, maternal ethnicity, and maternal education |
| Hultman et al. (2002) ^2^ | Sweden | Nested case-control | This case-control study nested in a population-based cohort defined by all infants born alive in Sweden from 1974 through 1993. The case sample was composed of 408 children registered in the Medical Birth Register who, at the age of 9 years or younger, had been discharged from a Swedish psychiatric or general hospital with a main diagnosis of infantile autism. | Boys: 4.4 years old  Girls: 4.6 years old | Maternal country of birth | Infantile autism (ICD-9 code 299A) | Maternal age, parity, smoking habits during pregnancy, hypertensive diseases, diabetes, pregnancy bleeding, mode of delivery, season of birth, gestational age (weeks), birth weight for gestational age, Apgar score at 5 minutes, congenital malformations |
| Lauritsen et al. (2005) ^3^ | Denmark | Cohort | The population-based cohort consisted of all children born in Denmark between 1 January 1984 and 31 December 1998. | Boys: 3 years old  Girls: 4 years old | Maternal country of birth | Childhood autism (ICD-10 code F84.0) or atypical autism (ICD-10 code F84.1) | Age, sex, interaction between age and sex, calendar period, maternal age, paternal age, maternal history of psychiatric disorder, paternal identity, paternal history of psychiatric disorder, history of psychiatric disorder in siblings, degree of urbanization of place of birth, maternal country of birth, parental countries of births |
| Maimburg et al. (2006) ^4^ | Denmark | Case-control | Cases were identified in the Danish Psychiatric Central Register. For each case, 10 controls were randomly selected from the Danish Civil Registration System. | 4.57 years old | Parent with foreign citizenship | Infantile autism (ICD-8 code 299.0 and ICD-10 code F84.0) | Mother and fathers age, mother’s citizenship, birthweight and gestational age, Apgar, birth defect and irregular foetal position. |
| Williams et al. (2008) ^5^ | Australia | Case-control | Electronic data were provided by the data custodian for the New South Wales (NSW) Midwives data collection, about all births in NSW from 1990 to 1999. Of the 368 children aged less than five reported to the study, 182 children were linked to NSW Midwives data. | NR | Maternal country of birth | Autistic Disorder (eligible children had at least one clinical criterion of the DSM-IV classification for Autistic Disorder) | Gender, gestation <37 weeks, maternal age >35 years |
| Keen et al. (2010) ^6^ | UK | Cohort | The study population was drawn from children presenting to the paediatric child development services of two adjacent south London boroughs, Wandsworth and Lambeth. | Lambeth Mean age (SD): 6.2 (2.4) years old  Wandsworth Mean age (SD): 4.6 (2.4) years old | Maternal region of birth | Autism, Other autism-spectrum disorders (diagnosed using Autism Diagnostic Interview (ADI-R), Diagnostic Interview for Social and Communication Disorders (DISCO) and Autism Diagnostic Observation Schedule (ADOS) reference to ICD-10) | Possible variation in rate of autism spectrum disorders resulting from differences in family size in different ethnic subgroups. |
| Haglund et al. (2011) ^7^ | Sweden | Case-control | All children suspected of having ASD are admitted to the Malmoe child psychiatric clinic. The control group was identified from the Swedish Medical Birth Registry (MBR), and consisted of all individuals who were born in Malmoe during the study period, and were not included in the case group. | NR | Maternal country of birth. | Autism diagnosed according to DSM-IV (earlier cases DSM-III) (Autistic Disorder, 29900), or ICD-10 (Childhood autism, F840); Asperger syndrome diagnosed according to DSM-IV (29980), ICD-10 (F985), or Gillberg and Gillberg criteria (1989). | Year of birth, maternal age at delivery, parity, maternal smoking in early pregnancy, gestational age-adjusted weight Standard Deviation-scores, and any obstetrical risk factor |
| Magnusson et al. (2012) ^8^ | Sweden | Nested case–control study | This matched case–control study nested within the Stockholm Youth Cohort, a register-based cohort of all children aged 0–17 years living in Stockholm County between 2001 to 2007. | NR | Parental country of birth | ASD diagnostic guidelines require the use of structured diagnostic assessments covering the child’s social, medical and developmental history, observation of the child in naturalistic settings and a structured neuropsychiatric assessment including cognitive testing using standardised and internationally recognised tools (such as Wechsler Intelligence Scale for Children, Wechsler Preschool and Primary Scale of Intelligence, Snijders-Oomen Non-Verbal Intelligence Test [Revised], and Leiter). | Maternal and paternal age at child’s birth and family disposable income at child’s birth or in early life |
| Lehti et al. (2013) ^9^ | Finland | Nested case–control study | Children born in 1987–2005 and diagnosed with childhood autism by the year 2007 were identified from the Finnish Hospital Discharge Register (FHDR), a nationwide register maintained by THL. Four controls per case were selected from the Finnish Medical Birth Register (FMBR), which is another mandatory national register maintained by THL. | 3.8 years old (both immigrant parents)  5.6 years old (both Finnish parents) | Parental country of birth | Autism (ICD-9 code 299.0 and ICD-10 code F84.0) | Parental age |
| Singh et al. (2013) ^10^ | US | Cross-sectional | This study based on the 2007 National Survey of Children’s Health (NSCH). The 2007 NSCH was a telephone survey conducted from April 2007 to July 2008. It had a sample size of 91,642 children <18 years of age, including a sample of about 1,800 children per state. | NR | Foreign-born children with both immigrant parents (first generation) and U.S.-born children with one or both immigrant parents (second generation). U.S.-born children with both U.S.-born parents (third or higher generation) were considered native-born. | ASD based on the questions, “Has a doctor or other health-care provider ever told you that the child had autism, Asperger’s Disorder, pervasive developmental disorder, or other autism spectrum disorder (ASD)? Does the child currently have autism or ASD?” ASD indicator is defined for children aged 3–17 years.  ADD/ADHD based on the questions, “Has a doctor or other health-care provider ever told you that the child had attention deficit disorder (ADD) or attention deficit hyperactivity disorder (ADHD)? Does the child currently have ADD or ADHD?” ADD/ADHD indicator is defined for children aged 2–17 years. | Child’s age, sex, race/ethnicity, household composition, metropolitan/non-metropolitan residence, household poverty, and education level |
| van der Ven et al. (2013) ^11^ | Netherlands | Cohort | Cohort consisting of all live births in the urban area of Utrecht and surrounding semirural municipalities between January 1, 1998 and December 31, 2007. The second source of the data originates from The Psychiatric Case Registry of the central part of the Netherlands (PCR-MN), which is in operation since 1999. It contains anonymized information on all patients who attended in- or out-patient facilities for mental health care until December 31st 2009, including date of birth, gender, postal code and at least one DSM-IV diagnosis. | Netherlands Mean age (SD): 6.3 (2.3) years old | Paternal country of birth | Autistic disorder (DSM-IV code 299.00), Asperger syndrome (299.80) or Pervasive Developmental Disorder Not Otherwise Specified (299.80) | gender and paternal age. |
| Becerra et al. (2014) ^12^ | U.S. | Cohort | Children born to mothers who resided in LA County, California, between 1995 and 2006. | 36–71 months of age | Parental country of birth | Autistic disorder (based on the DSM- IV-R and ICD-9-CM code 299.00), and as reported on the DDS Client Development Evaluation Report (CDER) | Maternal age (<18, 19–25, 26–30, 31–35, or.35 years), type of birth (single or twin+), parity (1, 2, 3, or >3 children), infant gender (male or female), year of birth (1995–2006), gestational age (<37 weeks or ≥37 weeks), birth weight (<2500, 2500–4500, or >4500 g), trimester start of prenatal care (no care, first, second, or third trimester), and any pregnancy complication (hypertension, renal, lung, or cardiac disease, asthma, pyelonephritis, diabetes, gestational diabetes, Rh sensitivity, hemoglobinopathy, uterine bleeding, hydramnios, incomplete cervix, sexually transmitted diseases, hepatitis B, rubella, other infections, prenatal tobacco use, and large fibroids) and maternal education (less than high school, high school, more than high school) and insurance type (Medi-Cal, private insurance, other) and Regional Centre |
| Abdullahi et al. (2019) ^13^ | Australia | Cohort | The retrospective cohort study included all children born alive in Western Australia (WA) from 1980 to 2010, using de-identified population-based data linked across health and disability datasets. The 4 database collections used in this study were the WA Midwives Notification System (MNS), the WA Birth Register, the Intellectual Disability Exploring Answers (IDEA) database, and the Western Australian Register of Developmental Anomalies (WARDA). | NR | Maternal country of birth | ASD with/without intellectual disability | P1 adjusted for sex, maternal age, IRSAD, birth year, and parity; P2 adjusted for sex, maternal age, IRSAD, birth year parity, and smoking. |
| **Risk of ADHD** |  |  |  |  |  |  |  |
| Huss et al. (2008) ^14^ | German | Cross-sectional | Nationwide, representative, cross-sectional health interview and examination survey with a total of 17,641 examined children and adolescents aged 0–17 years | NR | Children from families with a migration history | ADHD and potential ADHD (individuals reach a clinically significant score of 7 on the hyperactivity-inattention subscale of the SDQ and without a diagnosis by a medical doctor or psychologist) | Gender, socioeconomic status, history of migration, and age |
| Lehti et al. (2016) ^15^ | Finland | Nested case–control study | All singletons born in Finland in 1991–2005, those diagnosed with ADHD by the year 2011 were included in the study. Four controls per case were selected through linkage between the Finnish Hospital Discharge Register and the Finnish Central Population Register, which is a national register that contains basic information about Finnish citizens and permanent residents in Finland. | NR | Immigrant parents were defined as those who were born abroad and not native Finnish speakers. Those who were born in Finland and/ or whose native language was Finnish were defined as Finnish. | ADHD diagnosed based on ICD-9 codes 314.00, 314.01, 314.1, 314.2, 314.8, and 314.9 and ICD-10 codes F90.0, F90.1, F90.8, and F90.9 | Maternal age, paternal age, maternal smoking during pregnancy, parity, urbanization of birthplace, maternal marital status, and maternal socioeconomic status |
| Cotter et al. (2019) ^16^ | Ireland | Cohort | Using the Child cohort (Cohort’ 98) of the ‘Growing up in Ireland’ study, which randomly selected 900 schools in Ireland to recruit participants for the child cohort. | Wave 1: 9 years old  Wave 2: 13 years old | Children without Ireland citizenship | Hyperactivity (SDQ hyperactivity subscale scoring ≥ 90th percentile) | Socio-economic status and language spoken at home between the child and the primary care giver. |
| Osooli et al. (2021) ^17^ | Sweden | Cohort | Eligible participants were born 1987–2010 and were registered as residents of Sweden during at least one year at some time during the study period between Jan 1st 2001and Dec 31st 2015. | NR | Immigrants were defined as first- and second-generation immigrants. First-generation immigrants were those born abroad with both parents born abroad. Second-generation immigrants were born in Sweden and divided into three subgroups based on parental country of birth: 1) Both parents were foreign-born; 2) Swedish-born father and foreign-born mother; and 3) Swedish-born mother and foreign-born father. | ADHD (ICD-10 code F90) | Birth year and age and maternal income at baseline |
| **Hyperactive (SDQ)** |  |  |  |  |  |  |  |
| Leavey et al. (2004) ^18^ | U.K. | Cross-sectional | In September 2000, the study surveyed 11–16-year-old pupils attending one large secondary school in north London. | 11–16 years old | Children foreign born | Hyperactivity score (SDQ self-report version) | - |
| Derluyn et al. (2007) ^19^ | Belgium | Case-control | The study was carried out in intensive language classes for recently arrived, non-Dutch speaking migrant adolescents in Flanders (Belgium). Control group of Belgian adolescents was randomly selected form the five Flemish provinces in 17 schools for adolescents at same age. | 11-18 years old | Migrant status | Hyperactivity score (SDQ self-report version) | - |
| Holling et al. (2008) ^20^ | German | Cross-sectional | The KiGGS study is a nationwide representative cross-sectional health interview and examination survey among children and adolescents. The data was collected from May 2003 to May 2006 at 167 representatively selected sample points all over Germany. | 3-17 years old | Children with one or both immigrant parents (born outside the Germany) | Hyperactivity score (SDQ parent version) | - |
| Sagatun et al. (2008) ^21^ | Norway | Cohort | All 10th graders in Oslo during the school years 1999–2000 and 2000–2001 were invited to enter the youth part of the Oslo Health Study. | 15-16 years old (baseline) | Parental country of birth | Hyperactivity score (SDQ self-report version) | - |
| Alonso-Fernandez et al. (2017) ^22^ | Spain | Case-control | The SNHS were carried out on a representative sample of the non-institutionalized population of Spain. Children aged 0–14 years and adults aged 15 or more in the SNHS 2012 were chosen to complete the questionnaire. Two controls were randomly selected for each case, matched by age, gender, province and town size. | 4-14 years old | The subjects chose the “foreigner” option as an answer to the question “What is the nationality of…?” | Hyperactivity score (SDQ) | - |
| McMahon et al. (2017) ^23^ | Austria Estonia France Germany Hungary Ireland Italy Romania Slovenia Spain | Cross-sectional | Data were drawn from the Saving and Empowering Young Lives in Europe (SEYLE) study, a randomized controlled trial (RCT). Participants were recruited from 168 schools in 10 EU countries (Austria, Estonia, France, Germany, Hungary, Ireland, Italy, Romania, Slovenia and Spain). | Mean age (SD): 14.8 (0.84) years old | First-generation migrant children were born abord, second-generation migrant children were domestically born with foreign-born parents | Hyperactivity score (SDQ self-report version) | - |

**Abbreviations**: ADHD, attention-deficit/hyperactivity disorder; ASD, autism spectrum disorder; CI, confidence interval; DSM, Diagnostic and Statistical Manual of Mental Disorders; ICD, International Classification of Diseases; NOS, Newcastle–Ottawa scale; SD, standard deviation; SDQ, Strengths and Difficulties Questionnaire.

# Supplementary Table 3. Quality of cohort studies included in meta-analysis according to the Newcastle-Ottawa Scale

| Author (year) | Selection | | | |  | Comparability |  | Outcome | | | Overall score |
| --- | --- | --- | --- | --- | --- | --- | --- | --- | --- | --- | --- |
|  | Representativeness of the exposed cohort | Selection of the non-exposed cohort | Ascertainment of exposure | Demonstration that outcome of interest was not present at start of study |  | Comparability of cohorts on the basis of the design or analysis |  | Assessment of outcome | Was follow-up long enough for outcomes to occur | Adequacy of follow up of cohorts |  |
| Croen et al. (2002) ^1^ | 1 | 1 | 1 | 1 |  | 2 |  | 1 | 1 | 1 | 9 |
| Lauritsen et al. (2005) ^3^ | 1 | 1 | 1 | 1 |  | 2 |  | 1 | 1 | 1 | 9 |
| Sagatun et al. (2008) ^21^ | 1 | 1 | 1 | 1 |  | 1 |  | 1 | 0 | 0 | 6 |
| Keen et al. (2010) ^6^ | 0 | 1 | 1 | 1 |  | 1 |  | 1 | 1 | 1 | 7 |
| van der Ven et al. (2013) ^11^ | 1 | 1 | 1 | 1 |  | 1 |  | 1 | 1 | 1 | 8 |
| Becerra et al. (2014) ^12^ | 1 | 1 | 1 | 1 |  | 2 |  | 1 | 1 | 1 | 9 |
| Abdullahi et al. (2019) ^13^ | 1 | 1 | 1 | 1 |  | 1 |  | 0 | 1 | 1 | 7 |
| Cotter et al. (2019) ^16^ | 1 | 1 | 1 | 1 |  | 0 |  | 0 | 1 | 1 | 6 |
| Osooli et al. (2021) ^17^ | 1 | 1 | 1 | 1 |  | 2 |  | 1 | 1 | 1 | 9 |

# Supplementary Table 4. Quality of case-control/cross-sectional studies included in meta-analysis according to the Newcastle-Ottawa Scale

| Author (year) | Selection | | | |  | Comparability |  | Exposure | | | Overall score |
| --- | --- | --- | --- | --- | --- | --- | --- | --- | --- | --- | --- |
|  | Definition adequate | Representativeness of the cases | Selection of controls | Definition of controls |  | Comparability of cases and controls |  | Ascertainment of exposure | Same method of ascertainment for cases and controls | Non-Response rate |  |
| Hultman et al. (2002) ^2^ | 1 | 1 | 1 | 1 |  | 1 |  | 1 | 1 | 0 | 7 |
| Leavey et al. (2004) ^18^ | 1 | 0 | 1 | 1 |  | 1 |  | 1 | 1 | 0 | 6 |
| Maimburg et al. (2006) ^4^ | 1 | 1 | 1 | 1 |  | 1 |  | 1 | 1 | 0 | 7 |
| Derluyn et al. (2007) ^19^ | 1 | 1 | 1 | 1 |  | 1 |  | 1 | 1 | 0 | 7 |
| Holling et al. (2008) ^20^ | 1 | 1 | 1 | 1 |  | 0 |  | 1 | 1 | 1 | 7 |
| Huss et al. (2008) ^14^ | 1 | 1 | 1 | 1 |  | 1 |  | 1 | 1 | 0 | 7 |
| Williams et al. (2008) ^5^ | 1 | 0 | 1 | 1 |  | 1 |  | 1 | 1 | 0 | 6 |
| Haglund et al. (2011) ^7^ | 1 | 1 | 1 | 1 |  | 1 |  | 1 | 1 | 0 | 7 |
| Magnusson et al. (2012) ^8^ | 1 | 1 | 1 | 1 |  | 2 |  | 1 | 1 | 0 | 8 |
| Lehti et al. (2013) ^9^ | 1 | 1 | 1 | 1 |  | 1 |  | 1 | 1 | 1 | 8 |
| Singh et al. (2013) ^10^ | 0 | 1 | 1 | 1 |  | 2 |  | 1 | 1 | 0 | 7 |
| Lehti et al. (2016) ^15^ | 1 | 1 | 1 | 1 |  | 2 |  | 1 | 1 | 1 | 9 |
| Alonso-Fernandez et al. (2017) ^22^ | 1 | 1 | 1 | 1 |  | 1 |  | 1 | 1 | 0 | 7 |
| McMahon et al. (2017) ^23^ | 1 | 1 | 1 | 1 |  | 1 |  | 1 | 1 | 1 | 8 |

# Supplementary Table 5. Associations between migration status and hyperactive score (SDQ)

| **Groups** | **No. study** | **No. participant** | **SMD**  **(95% CIs)** | **P for Z test** | **Heterogeneity** | | **P for interaction** |
| --- | --- | --- | --- | --- | --- | --- | --- |
|  |  |  |  |  | **I^2^ (%)** | **P** |  |
| **Overall difference** | 6 ^18-23^ | 31,158 | -0.073 (-0.383, 0.236) | 0.642 | 98.1 | <0.001 |  |
| **Migration generation** |  |  |  |  |  |  | 0.545 |
| 1st generation | 4 ^18,19,22,23^ | 14,318 | -0.181 (-0.734, 0.372) | 0.522 | 98.0 | <0.001 |  |
| 2nd generation | 2 ^21,23^ | 13,373 | -0.005 (-0.143, 0.134) | 0.948 | 44.4 | 0.180 |  |
| **Influence analyses ^a^** |  |  |  |  |  |  |  |
| Minimal | - | 30,337 | -0.119 (-0.456, 0.218) | 0.331 | 79.9 | <0.001 |  |
| Maximal | - | 29,756 | 0.057 (-0.058, 0.172) | 0.489 | 98.4 | <0.001 |  |

**Note**: ^a^ Influence analysis was conducted by eliminating one study at a time; excluded study by Alonso-Fernandez et al. ^22^ for minimal pooled ORs, and excluded study by Derluyn et al. ^19^ for maximal pooled ORs.

**Abbreviations**: CI, confidence interval; SDQ, Strengths and Difficulties Questionnaire; SMD, standardized mean differences.

# Supplementary Fig. 1 Forest plot for standardized mean differences of hyperactivity score between migrant and non-migrant children


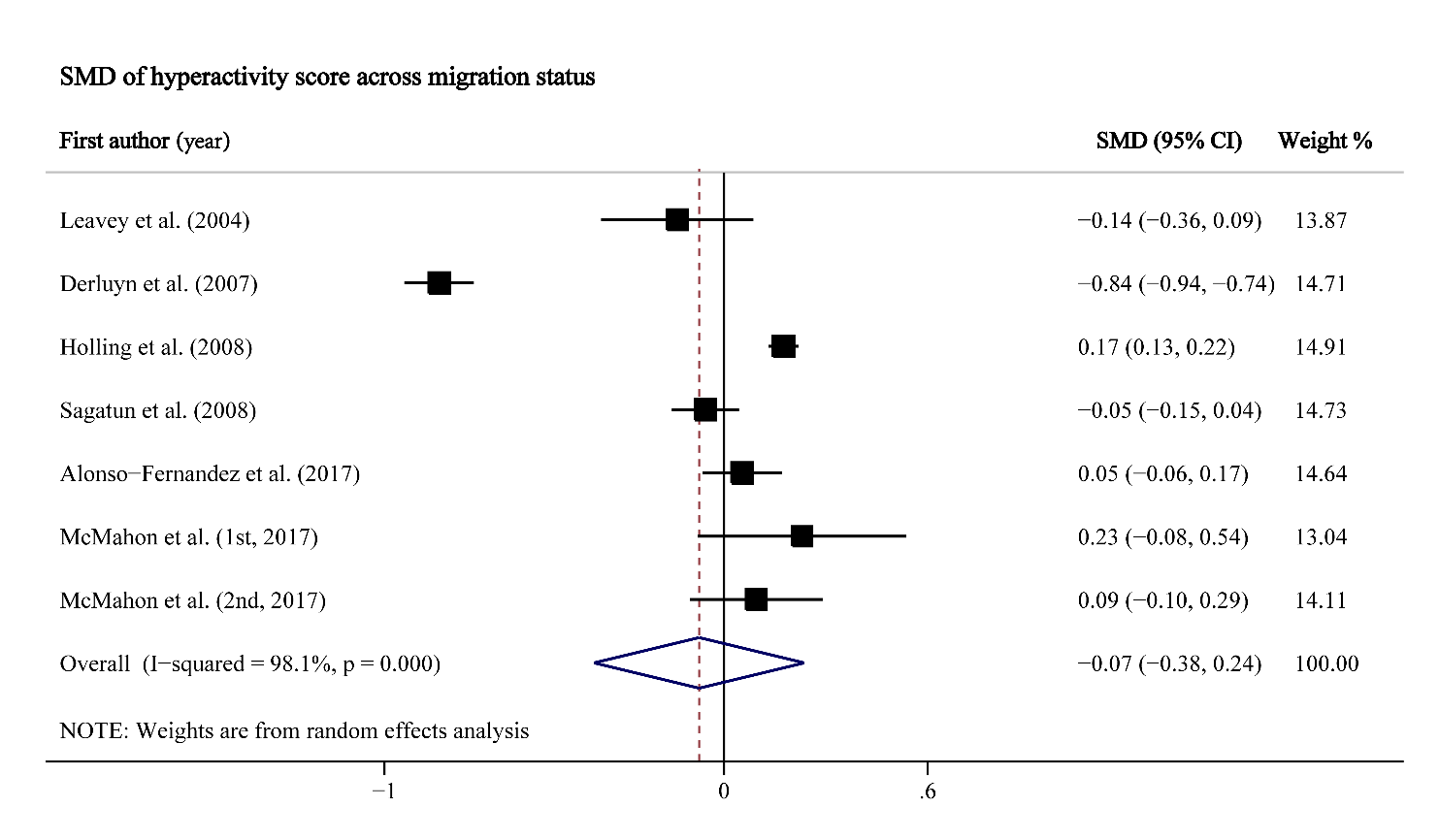


**Abbreviations:** CI, confidence interval; SMD, standardized mean differences.

# Supplementary Fig. 2 Funnel plot for studies evaluating risk of ASD among migrant children


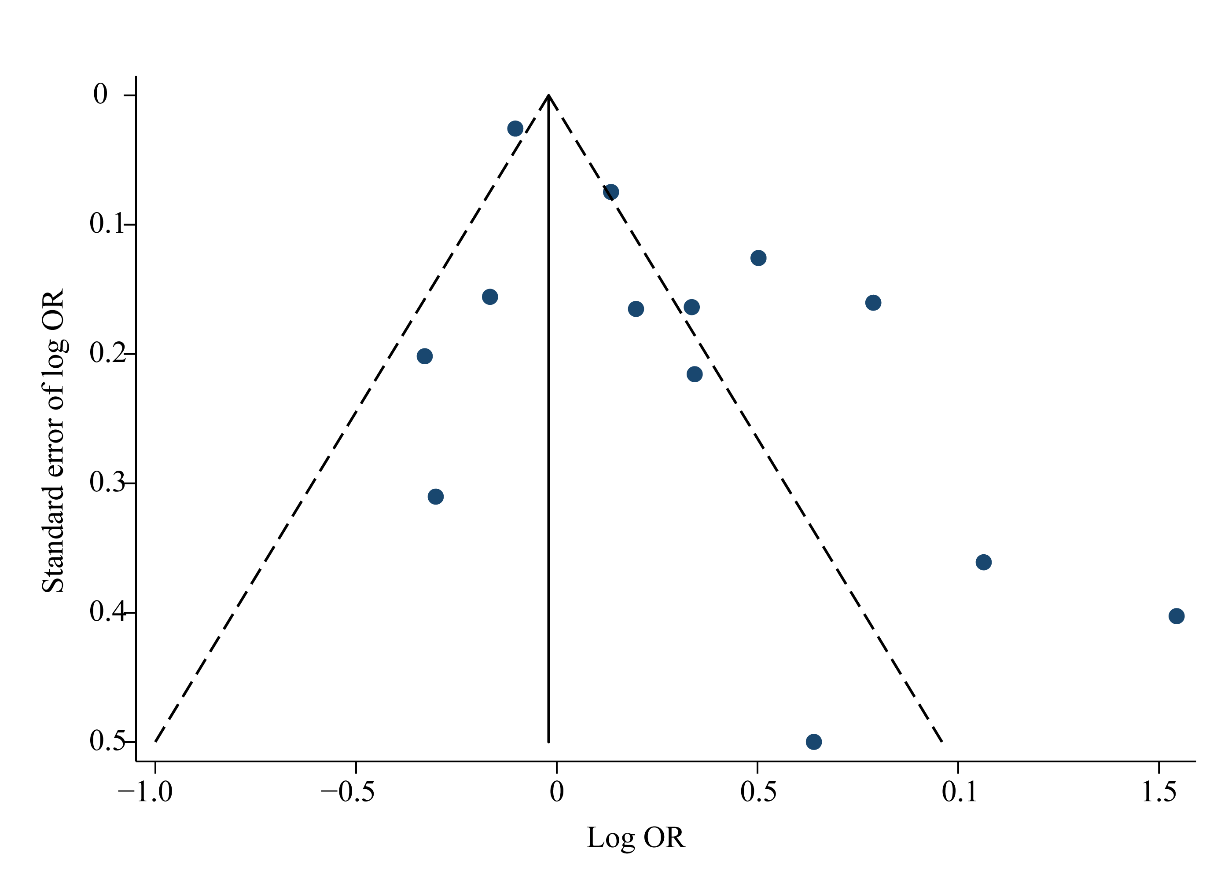


# Supplementary Fig. 3 Funnel plots for studies evaluating risk of ASD among migrant children using the trim-and-fill method


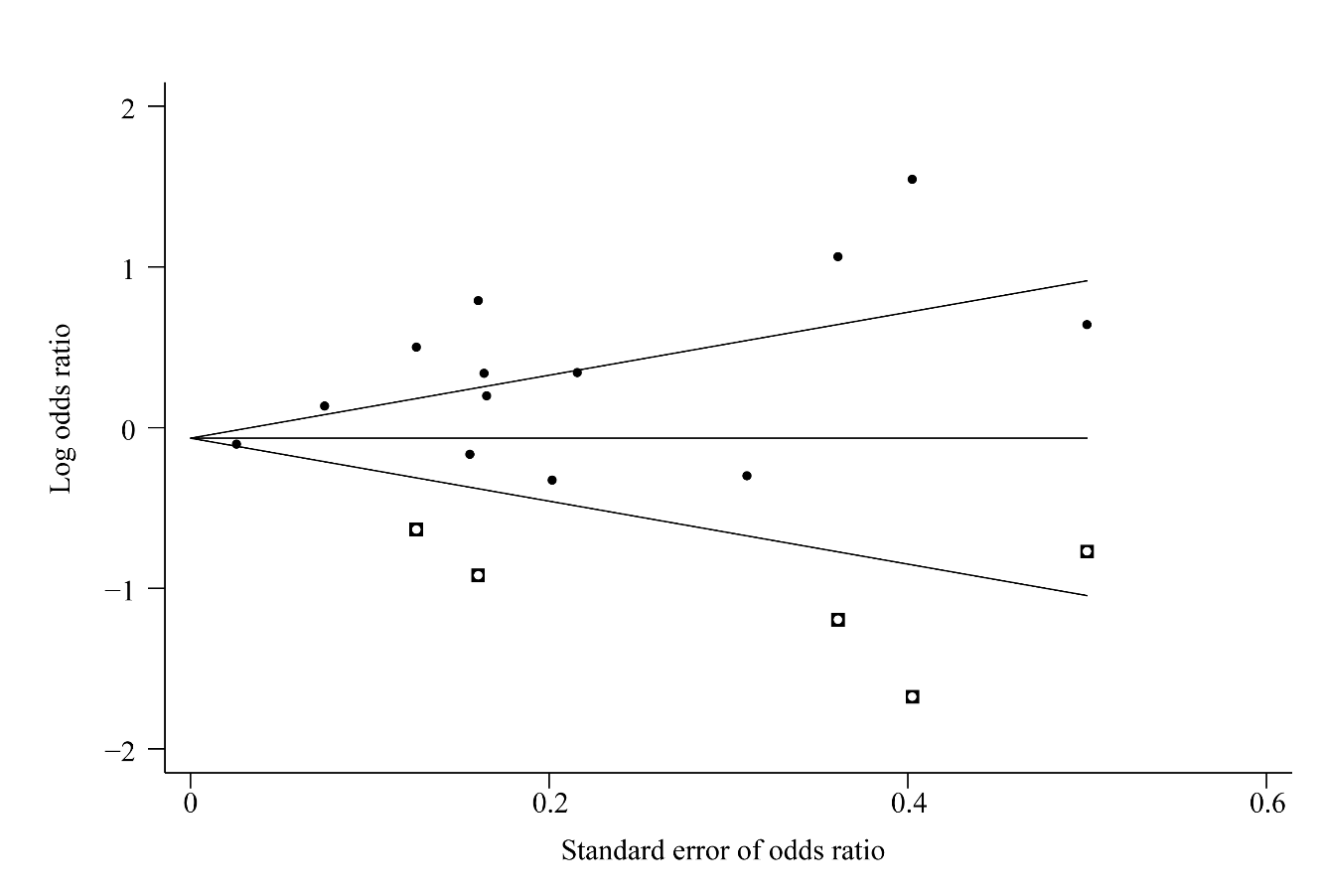


# Supplementary Fig. 4 Funnel plot for studies evaluating risk of ADHD among migrant children


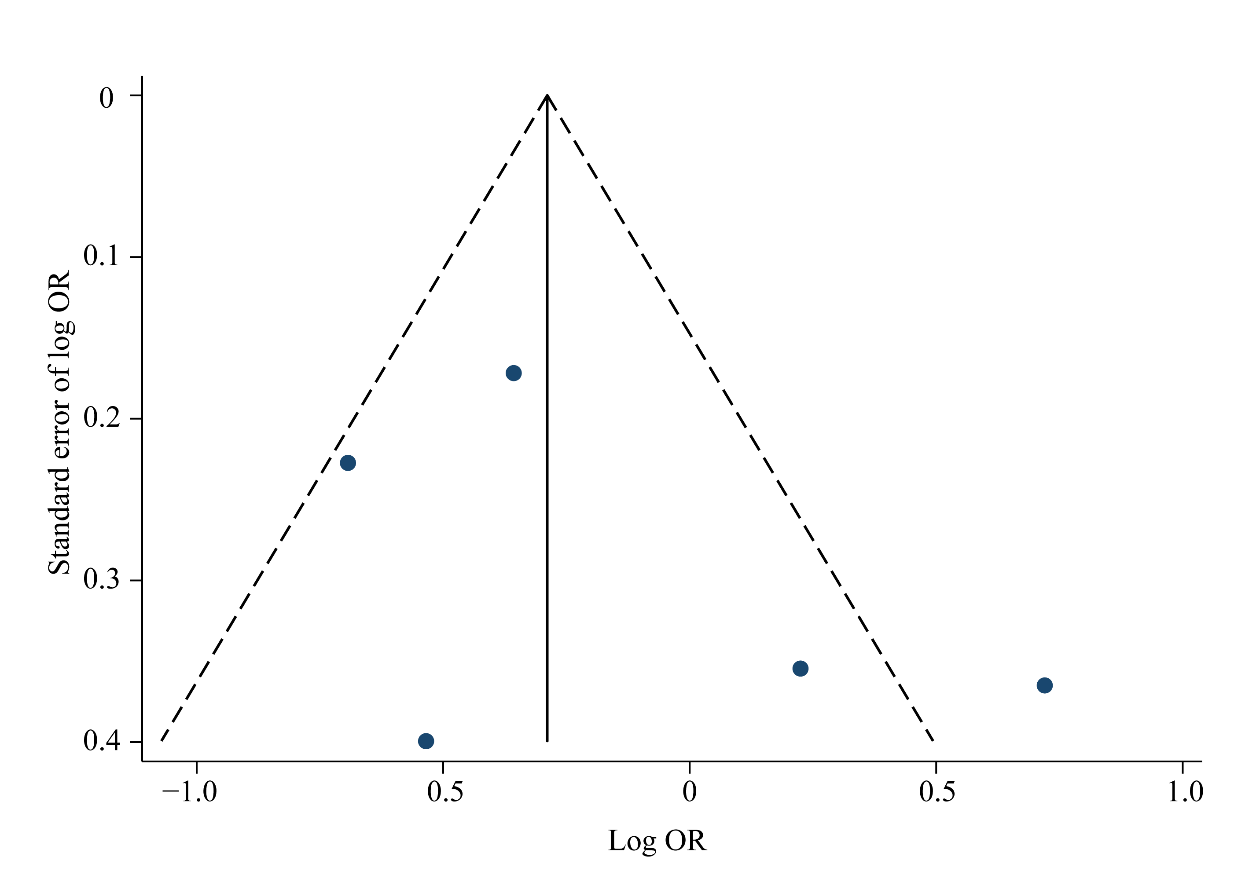


# References in supplementary material:

1. Croen LA, Grether JK, Selvin S. Descriptive epidemiology of autism in a California population: who is at risk? J Autism Dev Disord 2002; 32(3): 217-24.

2. Hultman CM, Sparén P, Cnattingius S. Perinatal risk factors for infantile autism. Epidemiology 2002; 13(4): 417-23.

3. Lauritsen MB, Pedersen CB, Mortensen PB. Effects of familial risk factors and place of birth on the risk of autism: a nationwide register-based study. Journal of child psychology and psychiatry, and allied disciplines 2005; 46(9): 963-71.

4. Maimburg RD, Vaeth M. Perinatal risk factors and infantile autism. Acta Psychiatr Scand 2006; 114(4): 257-64.

5. Williams K, Helmer M, Duncan GW, Peat JK, Mellis CM. Perinatal and maternal risk factors for autism spectrum disorders in New South Wales, Australia. Child Care Health Dev 2008; 34(2): 249-56.

6. Keen DV, Reid FD, Arnone D. Autism, ethnicity and maternal immigration. The British journal of psychiatry : the journal of mental science 2010; 196(4): 274-81.

7. Haglund NG, Kallen KB. Risk factors for autism and Asperger syndrome. Perinatal factors and migration. Autism : the international journal of research and practice 2011; 15(2): 163-83.

8. Magnusson C, Rai D, Goodman A, et al. Migration and autism spectrum disorder: population-based study. The British journal of psychiatry : the journal of mental science 2012; 201: 109-15.

9. Lehti V, Hinkka-Yli-Salomaki S, Cheslack-Postava K, Gissler M, Brown AS, Sourander A. The risk of childhood autism among second-generation migrants in Finland: a case-control study. BMC Pediatr 2013; 13: 171.

10. Singh GK, Yu SM, Kogan MD. Health, chronic conditions, and behavioral risk disparities among U.S. immigrant children and adolescents. Public Health Rep 2013; 128(6): 463-79.

11. van der Ven E, Termorshuizen F, Laan W, Breetvelt EJ, van Os J, Selten JP. An incidence study of diagnosed autism-spectrum disorders among immigrants to the Netherlands. Acta Psychiatr Scand 2013; 128(1): 54-60.

12. Becerra TA, von Ehrenstein OS, Heck JE, et al. Autism spectrum disorders and race, ethnicity, and nativity: a population-based study. Pediatrics 2014; 134(1): e63-71.

13. Abdullahi I, Wong K, Mutch R, et al. Risk of Developmental Disorders in Children of Immigrant Mothers: A Population-Based Data Linkage Evaluation. J Pediatr 2019; 204: 275-84.e3.

14. Huss M, Hölling H, Kurth BM, Schlack R. How often are German children and adolescents diagnosed with ADHD? Prevalence based on the judgment of health care professionals: results of the German health and examination survey (KiGGS). European child & adolescent psychiatry 2008; 17 Suppl 1: 52-8.

15. Lehti V, Chudal R, Suominen A, Gissler M, Sourander A. Association between immigrant background and ADHD: a nationwide population-based case-control study. Journal of child psychology and psychiatry, and allied disciplines 2016; 57(8): 967-75.

16. Cotter S, Healy C, Ni Cathain D, Williams P, Clarke M, Cannon M. Psychopathology and early life stress in migrant youths: an analysis of the 'Growing up in Ireland' study. Ir J Psychol Med 2019; 36(3): 177-85.

17. Osooli M, Ohlsson H, Sundquist J, Sundquist K. Attention deficit hyperactivity disorder in first- and second-generation immigrant children and adolescents: A nationwide cohort study in Sweden. Journal of Psychosomatic Research 2021; 141.

18. Leavey G, Hollins K, King M, Barnes J, Papadopoulos C, Grayson K. Psychological disorder amongst refugee and migrant schoolchildren in London. Social psychiatry and psychiatric epidemiology 2004; 39(3): 191-5.

19. Derluyn I, Broekaert E, Schuyten G. Emotional and behavioural problems in migrant adolescents in Belgium. European child & adolescent psychiatry 2008; 17(1): 54-62.

20. Holling H, Kurth BM, Rothenberger A, Becker A, Schlack R. Assessing psychopathological problems of children and adolescents from 3 to 17 years in a nationwide representative sample: results of the German health interview and examination survey for children and adolescents (KiGGS). European child & adolescent psychiatry 2008; 17 Suppl 1: 34-41.

21. Sagatun A, Lien L, Sogaard AJ, Bjertness E, Heyerdahl S. Ethnic Norwegian and ethnic minority adolescents in Oslo, Norway. A longitudinal study comparing changes in mental health. Social psychiatry and psychiatric epidemiology 2008; 43(2): 87-95.

22. Alonso-Fernandez N, Jimenez-Garcia R, Alonso-Fernandez L, Hernandez-Barrera V, Palacios-Cena D. Mental Health and Quality of Life Among Spanish-born and Immigrant Children in Years 2006 and 2012. J Pediatr Nurs 2017; 36: 103-10.

23. McMahon EM, Corcoran P, Keeley H, et al. Mental health difficulties and suicidal behaviours among young migrants: multicentre study of European adolescents. BJPsych open 2017; 3(6): 291-9.
